# AKT inhibitors in gynecologic oncology: past, present and future

**DOI:** 10.3389/fonc.2025.1547083

**Published:** 2025-07-17

**Authors:** Jinghong Chen, Rutie Yin

**Affiliations:** Department of Obstetrics and Gynecology, West China Second University Hospital, Key Laboratory of Birth Defects and Related Diseases of Women and Children, Ministry of Education, Sichuan University, Chengdu, Sichuan, China

**Keywords:** AKT inhibitor, gynecological cancer, targeted therapy, biomarker, clinical trial

## Abstract

The PI3K/AKT/mTOR pathway serves as a critical signaling nexus in cancer, with AKT acting as a central regulator of tumor cell proliferation, survival, metabolism, and therapy resistance. AKT inhibitors show promising but variable anti-tumor activity in preclinical and clinical studies. Currently, multiple classes of AKT inhibitors—PH domain competitors (perifosine), allosteric inhibitors (MK-2206), and ATP-competitive agents (AZD5363, GSK2110183, GSK2141795, and GDC-0068) are under development, with several agents in phase II/III trials. While early results demonstrated encouraging response rates and prolonged PFS in selected patients, significant challenges remain. The efficacy needs confirmation in larger trials, toxicities require better management, and resistance mechanisms demand further elucidation to guide optimal therapeutic strategies. This study systematically reviews recent AKTi research in gynecological cancers, aiming to provide a theoretical foundation for identifying potential biomarkers, overcoming drug resistance, and developing prognostic models. These insights may further facilitate the clinical translation of key therapeutic agents.

## 1 Introduction

**Figure 1:**
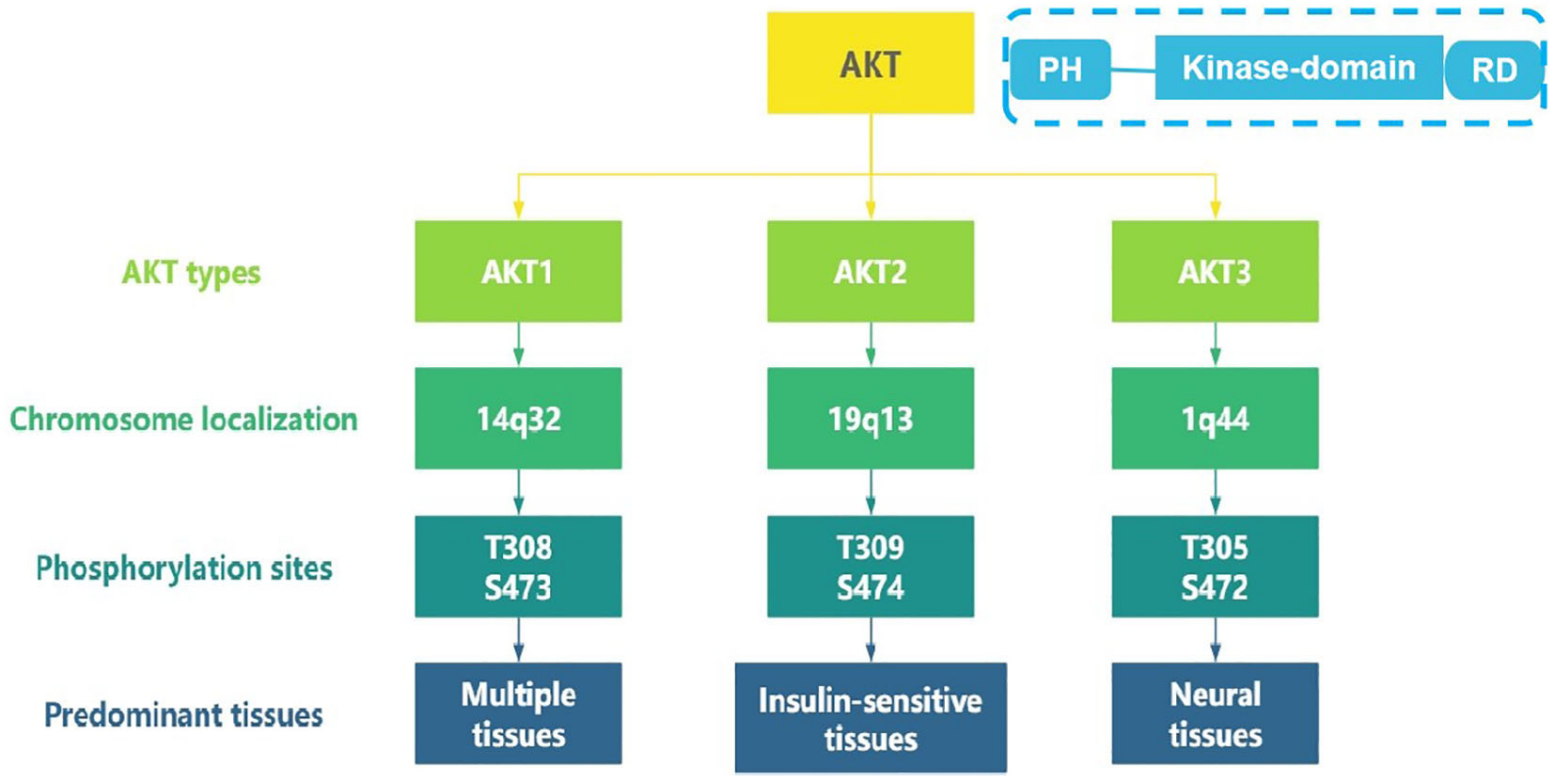
The different types of AKT and their characteristics.

As a central node in the phosphatidylinositol 3-kinase (PI3K)/AKT/mammalian target of rapamycin (mTOR) signaling pathway, the serine/threonine kinase AKT orchestrates diverse cellular processes including proliferation, survival, and metabolism (1). Three highly conserved isoform—*AKT1* (14q32.33), *AKT2* (19q13.2), and *AKT3* (1q43-q44), encoded by distinct chromosomal loci, share common structural features, with an N-terminal pleckstrin homology (PH) domain, a central kinase domain, and a C-terminal regulatory domain(see **Figure 1**). They exhibit isoform-specific functions due to differential tissue expression and interacting partners (2–4). AKT1 is ubiquitously expressed across multiple tissues and promotes cell survival primarily through its anti-apoptotic activity (5, 6). AKT2 is predominantly expressed in insulin-sensitive tissues, including brown adipose tissue, skeletal muscle, and the liver, where it plays a central role in mediating insulin-dependent glucose metabolism (6, 7). AKT3 demonstrates predominant expression in neural tissues and contributes to glioblastoma pathogenesis (8).

Hyperactivation of AKT signaling, achieved through gene amplification, somatic mutations, or upstream pathway alterations, represents a hallmark of malignant transformation (**Figure 1**). Amplification of *AKT1* gene represents a frequent oncogenic alteration across multiple tumor types. For example, AKT1 PH domain somatic mutations are found in 8.2% of breast cancers, 2% of ovarian cancers, and 5.9% of colorectal cancers (9). A large-scale multicenter study found that the overexpression of AKT2 was present in 12% of ovarian cancers (16/132) and 3% of breast cancers (3/106) (10). Additionally, *AKT2* amplification is more common in high-grade ovarian cancers with poor prognosis (10, 11). AKT3 overexpression is observed in 20% of ovarian cancer and 40% of primary melanoma (8, 12).

**Figure 2:**
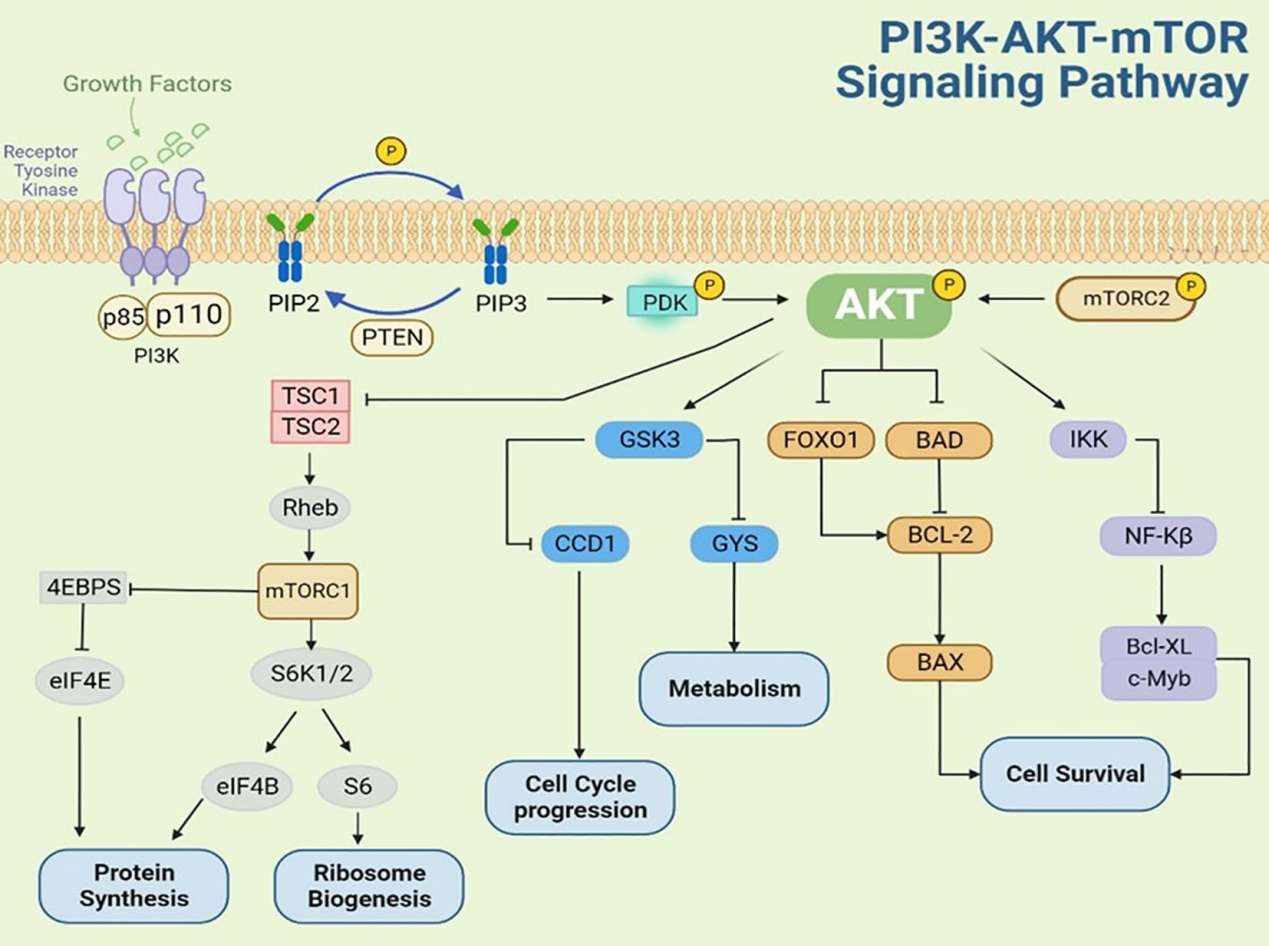
The molecular mechanisms of AKT regulation in PI3K/AKT/mTOR pathway.

AKT activation occurs through multiple mechanisms, including stimulation by growth factors such as fibroblast growth factor (FGF), vascular endothelial growth factor (VEGF), nerve growth factor, platelet-derived growth factor (PDGF), epidermal growth factor (EGF), insulin-like growth factor (IGF) (13). As shown in **Figure 2**, upon growth factor binding to receptor tyrosine kinases (RTKs), the PI3K regulatory subunit (p85) recognizes and binds to phosphotyrosine residues on the activated RTK cytoplasmic domain. This interaction recruits and activates the PI3K catalytic subunit (p110), leading to phosphatidylinositol-3,4,5-trisphosphate (PIP3) production at the plasma membrane. PIP3, acting as a second messenger, binds to the pleckstrin homology (PH) domain of AKT, inducing a conformational change that recruits AKT to the plasma membrane for complete phosphorylation. Following activation, phosphorylated AKT translocates to various cytoplasmic compartments where it propagates growth factor signals by phosphorylating downstream effectors, including p70 ribosomal S6 kinase 1 (p70S6K1) and eukaryotic translation initiation factor 4E-binding protein 1(4E-BP1). These phosphorylation events ultimately enhance protein translation, stimulate cell growth, and promote protein synthesis (14–18). The phosphatase and tensin homolog (PTEN), a tumor suppressor encoded on chromosome 10, functions as a critical negative regulator of the PI3K-AKT pathway by catalyzing the dephosphorylation PIP3 to PIP2, thereby attenuating PI3K-mediated signal transduction (19–21).

Therapeutic targeting of AKT has advanced through three inhibitor classes: PH domain competitors (perifosine), allosteric inhibitors (MK-2206), and ATP-competitive agents (GSK2110183, GSK2141795, GDC-0068, and AZD5363), which demonstrated significant anti-tumor efficacy in multiple cancer types across phase I-III clinical trials (22). The pan-AKT inhibitor AZD5363 (capivasertib) was firstly approved by FDA in November 2023 for use in combination with fulvestrant to treat HR-positive/HER2-negative breast cancer, which not only validates the AKT pathway as a druggable target, but also pioneers a new paradigm in biomarker-driven precision oncology (23). However, clinical trials of AKTi in gynecologic oncology—including ovarian cancer, endometrial cancer, and cervical cancer—remain limited (**Table 1**). This critical gap motivates our comprehensive review to synthesize the mechanistic rationale for AKT targeting across gynecologic cancers, overcome therapeutic resistance, and provide a roadmap for biomarker-driven clinical development - addressing an urgent unmet need in female-specific cancers.

**Table 1:** Clinical trials of AKTi in gynecologic oncology.

Drug	Year opened	Phase	Name	NCT	Disease	Treatment	Biomarker	Status	N	Primary objectives	Secondary objectives
Allosteric Akt inhibitor											
MK2206	2011	II	A Phase II, 2-Stage, 2-Arm PIK3CA Mutation Stratified Trial of MK-2206 in Recurrent or Advanced Endometrial Cancer	NCT01307631	Recurrent or Advanced Endometrial Cancer	Monotherapy	PIK3CAm	Completed	36	6-month PFS rate,CR,PR	PFS,OS,CTCAE4.0
	2011	II	A Phase II Study of MK-2206 in the Treatment of Recurrent High-Grade Serous Platinum-Resistant Ovarian, Fallopian Tube, or Primary Peritoneal Cancer	NCT01283035	Recurrent Platinum-Resistant Ovarian Cancer	Monotherapy	The frequency of mutations in the PI3K/AKT and RAS pathways, copy number alterations, and PTEN loss and AKT expression	Completed	6	ORR,Biomarker	Duration of PFS,toxities
ATP-competitive inhibitor											
Ipatasertib/RG7440/GDC-0068	2022	I/IB	Phase I/IB Safety and Pharmacodynamic Study of Neoadjuvant (NACT) Paclitaxel and Carboplatin With Ipatasertib as Initial Therapy of Ovarian Cancer PTMA 100805	NCT05276973	Ovarian Cancer	Monotherapy	PI3K pathway(PTEN,PIK3CA,PIK3R1,AKT1,p53 loss,KRAS,NF1,TSC1/TSC1)	Active, not recruiting	24	MTD,DLT,CTCAE5.0	ORR
	2021	IB/II	A Phase IB/II Multi-Cohort Study of Targeted Agents and/or Immunotherapy With Atezolizumab for Patients With Recurrent or Persistent Endometrial Cancer	NCT04486352	Endometrial Cancer	Combined therapy	PIK3CA/AKT1/PTEN-altered tumors	Recruiting	148	ORR	6-month PFS rate,DCR,DOR,OS
	2021	II	A Phase II, Open-Label, Multicenter, Platform Study Evaluating the Efficacy and Safety of Biomarker-Driven Therapies in Patients With Persistent or Recurrent Rare Epithelial Ovarian Tumors	NCT04931342	Ovarian Cancer ((PIK3CA/AKT1/PTEN-altered tumors))	Combined therapy	PIK3CA/AKT1/PTEN-altered tumors	Active, not recruiting	550	ORR	DOR,DCR,PFS,6-month PFS rate,OS
	2023	IB/II	A Phase IB and Randomized Phase II Trial of Megestrol Acetate With or Without Ipatasertib in Recurrent or Metastatic Endometrioid Endometrial Cancer	NCT05538897	Recurrent or Metastatic Endometrioid Endometrial Cancer	Combined therapy	—	Recruiting	96	toxity,RP2D,PFS	ORR,PK,biomarkers
Capivasertib/AZD5363	2010	II	A Phase I, Open-Label, Multicentre Study to Assess the Safety, Tolerability, Pharmacokinetics and Preliminary Anti-tumour Activity of Ascending Doses of AZD5363 Under Adaptable Dosing Schedules in Patients With Advanced Solid Malignancies	NCT01226316	Advanced or Metastatic Breast Cancer Ovarian Cancer Cervical Cancer Endometrial Cancer	Monotherapy	PIK3CAm,AKT1-E17Km	Completed	285	safety,PK	anti-tumour activity(RECIST1.1)
	2014	IB/II	A Phase Ib Study of the Oral PARP Inhibitor Olaparib With the Oral mTORC1/2 Inhibitor AZD2014 or the Oral AKT Inhibitor AZD5363 for Recurrent Endometrial, Triple Negative Breast, and Ovarian, Primary Peritoneal, or Fallopian Tube Cancer	NCT02208375	Endometrial, Triple negative Breast Cancer, Ovarian Cancer	Combined therapy	—	Active,not recruiting	159	MTD,RP2D	toxicities,6-month PFS rate,response duration,PK
	2014	I	A Phase I Multi-centre Trial of the Combination of Olaparib (PARP Inhibitor) and AZD5363 (AKT Inhibitor) in Patients With Advanced Solid Tumours	NCT02338622	Advanced Solid Tumours	Combined therapy	BRCAm	Completed	60	safety,MTD,RP2D	plasma levels,tumor biopsies
	2018	II	A Randomized Phase II Study Comparing Single-Agent Olaparib, Single Agent Cediranib, and the Combinations of Cediranib/Olaparib, Olaparib/Durvalumab (MEDI4736), Cediranib/Durvalumab (MEDI4736), Olaparib/AZD5363 (Capivasertib) in Women With Recurrent, Persistent or Metastatic Endometrial Cancer. A Multi-Arm Trial for Women With Recurrent or Persistent Endometrial Cancer	NCT03660826	Recurrent or Persistent Endometrial Cancer	Combined therapy	—	Active,not recruiting	288	PFS	OS,RR,safety
Uprosertib/GSK2141795	2009	I	A Phase I, Open-Label, Two-Stage Study to Investigate the Safety, Tolerability, Pharmacokinetics and Pharmacodynamics of the Oral AKT Inhibitor GSK2141795 in Subjects With Solid Tumors or Lymphomas	NCT00920257	Solid Tumors(Endometrial Cancer and Ovavrian Cancer)	Monotherapy	PTEN loss,PIK3CA	Completed	77	RP2D,safety\PK	clinical efficacy(RECIST1.1)
	2010	I	An Open Label Study To Investigate the Pharmacokinetics and Pharmacodynamics of Repeat Escalating Doses of the Oral AKT Inhibitor GSK2141795 by 18F FDG PET Analysis in Subjects With Ovarian Cancer	NCT01266954	Ovarian Cancer	Monotherapy	—	Completed	36	The amount of GSK2141795 in the blood (ng/ml) from baseline	The net unidirectional uptake of FDG (Ki) from baseline,The change in size of tumor from baseline (RECIST Criteria)
Afuresertib/GSK2110183/LAE002	2012	I/II	An Open-Label Phase I/II Study of GSK2110183 in Combination With Carboplatin and Paclitaxel in Subjects With Platinum-Resistant Ovarian Cancer	NCT01653912	Platinum-Resistant Ovarian Cancer	Combined therapy	—	Completed	59	CTCAE,MTD,ORR,	RR,PFS,etc.
	2020	II	An Open Label Randomized Active Controlled Phase II Clinical Study to Assess the Efficacy and Safety of Afuresertib Plus Paclitaxel Versus Paclitaxel in Patients With Platinum-Resistant Ovarian Cancer	NCT04374630	Platinum-Resistant Ovarian Cancer	Combined therapy	PI3K/AKT/PTEN pathway alterations,BRCA1/2m,level of pAKT	Completed	141	PFS	OS,ORR,DOR,DCR,BOR

## 2 AKTi and endometrial cancer

### 2.1 Preclinical research of AKTi in endometrial cancer

Alterations in the PI3K-AKT-mTOR pathway occur in 80-95% of endometrial cancer, representing the highest incidence among all solid malignancies. The expression of PI3K and AKT in endometrial cancer is higher than in normal endometrial tissue, and levels of expression are related to clinical staging, degree of diffentiation and prognosis (24–26).

AZD5363 (capivasertib) is a classic ATP competitive inhibitor. Preclinical studies have demonstrated its antitumor activity across 182 solid and hematologic tumor cell lines. In endometrial cancer cell lines, AZD5363 inhibits AKT, reduces phosphorylation of GSK3 and ribosomal protein S6, and consequently disrupts glucose metabolism and protein synthesis (27). Additionally, AZD5363 induces nuclear localization of forkhead box O1(FOXO1) and p53, reduces phosphorylation of the BCL2-associated agonist of cell death (BAD), disrupts cell cycle progression, and promotes apoptosis. In combination studies, AZD5363 synergistically enhances the cytotoxicity of doxorubicin in ECC-1 and A2780-CP drug-resistant cell lines (27). GDC-0068 (ipatasertib) is a potent and selective oral AKTi that has demonstrated significant antitumor efficacy in both preclinical models and clinical trials across multiple solid tumor types (28). GDC-0068 demonstrates dose-dependent inhibition of cell proliferation and colony formation in uterine serous carcinoma (USC) models. Notably, when compared with paclitaxel, GDC-0068 exhibits synergistic anti-tumor effects, significantly enhancing growth suppression and cleaved caspase-3 activation compared to single-agent treatment. These preclinical findings support GDC-0068 as a promising targeted therapy for USC, with clinical validation ongoing in multiple trials(NCT05538897, NCT04486352, NCT04931342). In PTEN-mutated endometrial cancer, hyperactivated AKT signaling transcriptionally suppresses progesterone receptor B (PRB), resulting in impaired progesterone responsiveness. The combination of MK-2206 with the progesterone receptor agonist R5020 effectively inhibits AKT activity and restores stable PRB expression in Ishikawa cells. Additionally, with the analysis to AKT-regulated PRB target genes, angiogenesis is mainly regulated by AKT-PRB. *In vitro*, the combination of MK2206 and R5020 significantly suppresses endometrial cancer epithelial cell invasion and vasculogenic capacity compared to monotherapy with either agent alone (29). A study evaluated the inhibitory effect of MK2206 on the growth and invasion of patient-derived xenograft (PDX) models of endometrial cancer. Three PDX cell lines—USC1, EEC2, and EEC4—were transplanted under the renal capsule of NSG mice. Two weeks post-engraftment, mice were administered either drug-loaded carriers (control) or MK2206 (120 mg/kg) twice weekly for three consecutive weeks. Compared to the control group, MK2206 treatment significantly suppressed tumor growth across all three PDX models, demonstrating its broad efficacy against different endometrial cancer subtypes. Histological analysis showed that the invasion and spreading of EEC2 and EEC4 tumors were significantly weakened after MK2206 treatment (30). The expression of procollagen-lysine, 2-oxoglutarate, 5-dioxygenase 2 (PLOD2) is upregulated in endometrial cancer cells under hypoxic conditions. This upregulation promotes cell migration, invasion, and epithelial-mesenchymal transition (EMT) via activation of the PI3K/AKT signaling pathway, which could be reversed by AKTi MK2206 (31).

Additionally, AKT-induced NF-κB activation critically contributes to estrogen-mediated angiogenesis, promoting the proliferation and clonogenic potential of endometrial cancer cells. AKTi effectively blocked estrogen-induced NF-κB activity, thereny suppressing tumorigenesis and progression in endometrial cancer (32). Insulin-like growth factor binding protein 2(IGFBP2) is overexpressed in endometrial cancer tissue and acted through the PI3K/AKT/mTOR pathway. Both the mTOR inhibitor RAD001 and the AKT inhibitor terameprocol (M4N) downregulated IGFBP2 expression in endometrial cancer cells by targeting the Sp1 transcription factor, resulting in synergistic inhibition of tumor growth (33). SIX1 overexpression upregulates cyclin D1, cyclin E, ERK, and AKT expression, enhancing tumor growth and colony formation capacity. Both the ERK inhibitor U0126 and AKT inhibitors effectively blocked SIX1-mediated proliferative effects (34). Although AKTis have demonstrated promising anti-tumor efficacy in preclinical models of endometrial cancer, its translational potential requires further validation in well-designed, rigorously controlled clinical trials.

### 2.2 Clinical trials of AKTi in endometrial cancer

AKTis have demonstrated limited clinical efficacy as monotherapy in patients with endometrial cancer. MK-2206 is an allosteric inhibitor of AKT with great activity against all three AKT isoforms, but the activity is most pronounced against AKT1 and AKT2 (35). Phase II trials of MK-2206 reported modest response rates and short median PFS, irrespective of PIK3CA mutation status (36, 37). A phase II study (NCT01307631) of MK-2206 in recurrent endometrial cancer (excluding carcinosarcoma) enrolled 36 patients, of whom 9 had PI3KCA gene mutation (see **Table 1**) (36). The overall cohort demonstrated a median PFS of 2.0 months (1.7m vs 2.5 m for *PIK3CA*-mutant vs wild-type, respectively) and median OS of 8.4 months (8.4m vs 11.1m, respectively).Common toxicities included rash (44%), fatigue (41%), nausea (42%), and hyperglycemia (31%). Notably, the MK-2206-treated cohort exhibited an unanticipated toxicity profile, with endometrial cancer patients demonstrating greater treatment-related adverse events (AEs) relative to other solid tumor populations. The pathophysiological basis for this tumor-specific toxicity enhancement remains undetermined. Another phase II study evaluated the use of MK-2206 in patients with advanced or recurrent high-grade serous endometrial cancer who had received more than two prior lines of therapy (37). Among 14 evaluable patients, one achieved confirmed partial response (PR), while two (including the PR patient) remained progression-free at 6 months. Five patients (35.7%) had stable disease (SD) lasting less than 6 months, seven (50%) experienced disease progression (PD), and one was not evaluable. The clinical benefit rate was 14.3% (95% CI 1.8%-42.8%). The most common AEs were diarrhea (36%), acneiform rash (36%), nausea (29%), fatigue (29%), and hyperglycemia (21%), most of which were grade 1-2.

GSK2141795 (uprosertib) is an effective ATP-competitive pan-AKTi, which reduces the phosphorylation of multiple AKT substrates and inhibits signaling in various cancer cells (38). In the NCT00920257 dose-escalation study (n=77, 12 with endometrial/uterine tumors and 9 with ovarian cancer), GSK2141795 showed favorable pharmacokinetics (t_1_/_2_=2.8 days) with the maximum tolerated dose (MTD) and the recommended phase II dose (RP2D) as 75mg QD (39). Among gynecologic malignancies, treatment-related AEs were diarrhea, fatigue, vomiting, and decreased appetite, while grade ≥3 hyperglycemia occurred in only 4% of the total cohort. The therapeutic potential of AKTi MK-2206 and GSK2141795 as single agents in endometrial cancer is restricted by insufficient efficacy and/or unacceptable toxicity profiles, highlighting the imperative for rationally designed combination therapies.

Emerging evidence revealed that AKTi reversed PARPi resistance through a novel synthetic lethality mechanism. PARPi-induced PI3K pathway upregulation is counteracted by AKTi, which attenuate homologous recombination repair capacity via BRCA1/2 downregulation. This dual-action strategy generates irreparable DNA damage and resensitizes tumors to PARP inhibition (40, 41). The phase I trial NCT02208375 investigated olaparib (300 mg twice daily) combined with AZD5363 (320 mg or 400 mg twice daily, 4-days-on/3-days-off schedule) in 38 patients with advanced/recurrent endometrial cancer (n=11, all BRCA-wild-type), ovarian cancer (n=16; 27% germline BRCA-mutated, 87% platinum-resistant), and triple-negative breast cancer (n=11) (42). At the RP2D (400 mg BID daily), the combination demonstrated an overall objective response rate of 19% (95% CI 7.2–36.4%) and clinical benefit rate of 41%, with differential activity observed across tumor types: endometrial cancer showed the highest response rate (ORR 44%, CBR 56%), followed by ovarian cancer (ORR 7%, CBR 43%; 83% of responders were platinum-resistant). With a median follow-up of 7.4 months (range 0.7–37.2), the regimen exhibited manageable toxicity without unexpected safety signals at the RP2D, supporting its further exploration in biomarker-selected populations. In a separate phase I trial (NCT02338622), Yap et al. (43) established two RP2D regimens for olaparib (300 mg BID) combined with AZD5363: either 400 mg BID (4 days on/3 off) or 640 mg BID (2 days on/5 off). Among 56 evaluable patients with diverse solid tumors, 44.6% achieved clinical benefit (CR/PR or SD≥4 months), including both *BRCA1/2*-mutant and wild-type tumors regardless of DDR or PI3K-AKT pathway status. Mechanistically, AKT inhibition significantly reduces pSer9-GSK3β and BRCA1 expression while increasing pERK, providing biological rationale for the observed synergy between these agents. Future clinical trials should address the critical unmet need of overcoming PARPi resistance in *BRCA* wild-type tumors.

## 3 AKTi and ovarian cancer

### 3.1 Preclinical research of AKTi in ovarian cancer

Among gynecologic malignancies, ovarian cancer carries the most dismal prognosis due to its propensity for recurrent relapses that progressively develop therapeutic resistance. This clinical challenge has intensified the focus on biomarker-guided precision therapy to improve survival and quality of life in recurrent disease. The PI3K-AKT-mTOR pathway serves as the metabolic-proliferative hub in ovarian cancer, driving tumorigenesis by orchestrating cell survival, cell cycle progression, and DNA repair. Critically, its aberrant activation, such as *PIK3CA* mutations and PTEN loss, not only directly promotes chemotherapy resistance and metastatic propensity, but also displays striking histotype-specific prevalence, which occur most frequently in ovarian clear cell carcinoma (OCCC; 20-46% and 20%, respectively) and endometrioid carcinoma (12-20% and 40%, respectively), but are rare in high-grade serous ovarian cancer (HGSOC; 2.3-3.7% and 7%, respectively) (44, 45). The histotype-specific activation patterns of PI3K-AKT-mTOR pathway not only predict therapeutic potential but also inform rational combination strategies to overcome acquired resistance (46).

A study using tumor samples to established Mini-PDX and PDX models from five PARPi-resistant, platinum-refractory ovarian cancer patients (including biopsy, surgical, and ascites specimens) demonstrated that 40% (2/5) responded to AKTi (uprosertib) monotherapy in Mini-PDX assays (47). In the PDX model, inhibition of AKT further enhanced the response of tumor cells to olaparib. Additionally, synergy was observed in PARP1-overexpressing cell lines(OVCA433, OVCAR8, and A2780) in the combination of uprosertib and olaparib. A preclinical study of W et al. suggested that MK-2206 inhibited AKT phosphorylation in *BRCA2* mutated cancer cells *in vitro*, making them more sensitive to cisplatin and olaparib (48). Additionally, AKTis showed multifaceted therapeutic synergy in ovarian cancer preclinical research. CircPLEKHM3 is a tumor suppressor that inhibits cell proliferation (49). CircPLEKHM3 binds to miR-9, enhancing the endogenous inhibitory effects of BRCA1, DNAJB6, and KLF4, which leads to the AKT1 signaling inactivation. The combination of paclitaxel and MK2206 exhibited synergistic effects in CircPLEKHM3-deficient cells, potentiating paclitaxel-induced inhibition of ovarian cancer cell growth. ^49 A^ preclinical study evaluated the AKT inhibitor SC66 in NOD-SCID xenograft models and eight ovarian cancer cell lines. SC66 effectively suppressed AKT phosphorylation and downstream signaling (4EBP1/p70S6K inhibition), while concurrently reducing expression of metastasis-associated TWIST1 and anti-apoptotic Mcl-1. Notably, SC66 resensitized chemotherapy-resistant cells to cisplatin and paclitaxel and significantly increased apoptosis rates (50).

Moreover, multiple novel AKTis are currently under development. Preclinical results showed that GSK2110183(afuresertib) showed a dose-dependent effect on multiple AKT substrate phosphorylation levels, including GSK3β, PRAS40, FOXO, and caspase 9. Overall, 65% of hematological cell lines were sensitive to GSK2110183(EC50<1 μmol/L). Among the solid tumor cell lines tested, 21% responded to GSK2110183 (EC50<1 μmol/L) (38). ARQ092 is an AKT allosteric inhibitor targeting the *E17K* hotspot mutation in AKT1, effectively suppressing AKT phosphorylation. Preclinical studies demonstrate its dual functionality in inhibiting ovarian cancer cell proliferation and chemosensitization, though clinical validation remains pending (51, 52). Isoalantolactone (IL) is one of the main constituents of Chrysanthemum, which has significant biological activity (53). IL induces AKT inactivation, reduces Bcl-2 protein expression, and triggers ovarian cancer cell apoptosis through cell cycle arrest and activation of downstream apoptosis-related molecules such as PARP-1 and caspase-3. In AKT-overexpressing SKOV-3 cells, IL combined with the AKTi wortmannin enhanced growth suppression, which was partially attenuated by acetylcysteine pretreatment. Ubiquitin specific peptidase 13 (USP13) is a key regulatory factor driving ovarian cancer metabolism, and silencing *USP13* significantly inhibits cell proliferation (54). Co-amplification of *USP13* with *PIK3CA* in the *3q26.3* was observed in 29.3% of HGSOC, which was significantly associated with poor clinical outcome. Inhibition of USP13 significantly suppressed tumor progression and sensitized tumor cells to PI3K/AKT inhibitors. While these preclinical studies demonstrated the therapeutic potential of AKTis, clinical translation requires further validation through large multicenter clinical trials.

### 3.2 Clinical trials of AKTi in ovarian cancer

Perifosine is a phospholipid analog that can target the PH domain of AKT and block the binding of PIP3 to the PH domain of AKT, thereby preventing AKT activation by preventing its translocation to the cell membrane (55). Additionally, it can inhibit the proliferation of ovarian cancer cells and enhance their sensitivity to paclitaxel (56) A phase II multicenter clinical trial (Japic CTI-132287) evaluated the efficacy and safety of perifosine monotherapy in the treatment of ovarian cancer, endometrial cancer, and cervical cancer. (57) A total of 71 patients (21 with ovarian cancer, 24 with endometrial cancer, and 26 with cervical cancer) were included in the study, and patients with recurrent or persistent ovarian, endometrial, and cervical cancer were divided into *PIK3CA-wt* and *PIK3CA-m* groups. All patients received 600 mg oral perifosine on day 1, followed by a maintenance dose of 100 mg per day. The results showed that the disease control rate (DCR) was 12.5% and 40.0% in patients with *PI3KCA-wt* and *PIK3CA-m* in ovarian cancer, 47.1% and 14.3% in endometrial cancer, 11.1% and 25.0% in cervical cancer. There were no significant differences in PFS and OS between *PI3KCA-wt* and *PIK3CA-m* in the three kinds of cancers. The most common grade 3/4 toxicities were anemia (22.5%) and anorexia (11.3%). That is, perifosine monotherapy were tolerated but the efficacy has not reached the expected level. Except for perifosine, no other AKTis targeting the PH domain have progressed into clinical research.

Preclinical studies have found that the sensitivity of AZD5363 was closely related to *PIK3 mutation* as well as the presence of other PI3K/AKT/mTOR pathway inhibitors (58–60). However, clinical studies of AZD5363 in gynecologic cancers have faced significant challenges. The First in Human trial (NCT01226316) demonstrated that AZD5363 was well-tolerated at the RP2D (480 mg BID, 4/3d), achieving significant plasma levels and potent target regulation in tumors (61). Notably, this trial was the first to evaluate biomarker-stratified cohorts (*PIK3CA*-mutant breast and gynecologic cancers) in patients treated with AKTi. While the study showed tumor volume reduction in 46% of breast cancer patients and 56% of gynecologic cancer patients, the ORR (4% and 8%, respectively) fell significantly below the predefined threshold of 20%. Consequently, further enrollment of *PIK3CA*-mutant patients was discontinued. Another subsequent study evaluated the safety and efficacy of AZD5363(480 mg BID 4/3d) in 58 patients including ER-positive breast cancer, and gynecologic malignancies(NCT01226316) (62). There were 52 patients with *AKT1-E17K-m*, 5 with *non-AKT-E17K-m*, and 1 of unknown mutation status. The mPFS for the ER-positive breast cancer, gynecologic cancer, and other solid tumor cohorts with *AKT1-E17K-m* (N=20, 15 and 17) were 5.5 months (95% CI, 2.9-6.9), 6.6 months (95% CI, 1.5-8.3), and 4.2 months (95% CI, 2.1-12.8), respectively. The ORR of 24% (0% in ovarian cancer, 25% in endometrial cancer) proved inferior to established targeted therapies for *EGFR*, ALK, *ROS1* or *BRAF*-mutant tumors. The safety profile was manageable, with grade 3 hyperglycemia (24%), diarrhea (17%), and maculopapular rash (15.5%) representing the most common toxicities. These findings suggest that while AZD5363 monotherapy shows limited efficacy in *AKT1/PIK3CA*-altered cancers, its potential may be better realized through rational combination strategies, warranting further clinical investigation (63–65).

In a phase I clinical trial, 11 out of 25 advanced ovarian cancer patients achieved clinical benefits from the combination therapy of AZD5363 and olaparib, as evidenced by RECIST criteria with CR, PR or SD lasting ≥4 months (43). Among the 25 patients with EOC, 5 had previous exposure to PARPi and subsequently developed resistance. Importantly, one of these PARPi-resistant patients achieved PR to the combination therapy. GSK2110183 (afuresertib) is an ATP-competitive AKTi that significantly delays tumor growth in human xenograft models. A phase I clinical study (NCT01653912) evaluated the efficacy and safety of GSK2110183 combined with paclitaxel and carboplatin in patients with recurrent or primary PROC (66). The first part was a dose-escalation study of the combination therapy for recurrent ovarian cancer (N=29). Patients received daily oral GSK2110183 (50–150 mg) in combination with intravenous paclitaxel (175 mg/m²) and carboplatin (AUC 5) every 3 weeks for 6 cycles, followed by maintenance GSK2110183 monotherapy (125 mg/day) until disease progression or unacceptable toxicity. The second stage employed a single-arm design to assess clinical activity of the combination regimen in recurrent or primary PROC (N=30). During the dose-escalation phase, three DLTs of grade 3 rash were observed (one at 125 mg/day and two at 150 mg/day), establishing 125 mg/day as the maximum tolerated dose (MTD) for GSK2110183 in combination with paclitaxel/carboplatin. In the efficacy evaluation cohort (n=30), the regimen demonstrated an ORR of 32% (95% CI 15.9-52.4) by RECIST 1.1 criteria and a GCIG CA125 response rate of 52% (95% CI 31.3-72.2), with a median PFS of 7.1 months (95% CI 6.3-9.0). In the phase II open-label randomized trial (NCT04374630, N=150) evaluating afuresertib combined with paclitaxel versus paclitaxel monotherapy in PROC patients, the addition of afuresertib failed to demonstrate statistically significant improvements in either PFS or OS (67). The experimental arm (afuresertib-paclitaxel) achieved median PFS of 4.3 months compared to 4.1 months with paclitaxel alone (HR 0.7, 95% CI 0.50-1.10; P=0.139), while median OS was 11.2 months versus 13.1 months (HR 1.2, 95% CI 0.77-1.81). These results indicate absence of meaningful clinical benefit in an unselected PROC population. However, biomarker-stratified analysis revealed significant PFS improvement in phospho-AKT-positive patients, with median PFS extending to 5.4 months in the experimental arm versus 2.9 months in controls, representing a 60% reduction in progression or death risk.

These studies provide compelling clinical evidence supporting the feasibility of combining AKT inhibition with conventional therapies, while underscoring the need to explore optimized combination regimens in biomarker-selected patient populations that demonstrated enhanced treatment efficacy.

## 4 AKTi and cervical cancer

Persistent HPV infection drives cervical carcinogenesis through E6/E7-mediated AKT pathway activation, promoting immune evasion and malignant transformation. AKT inhibition blocks this oncogenic process, suggesting therapeutic potential in cervical cancer (68, 69). Isoliensinine induced cervical cancer cell cycle arrest and apoptosis by down-regulating AKT (S473) phosphorylation and GSK3α expression through inhibition of the AKT/GSK3α pathway. The anti-tumor effects of isoliensinine were significantly enhanced when combined with the AKT inhibitor AKTi-1/2, demonstrating a synergistic therapeutic strategy for cervical cancer treatment (70). The *GADD45* gene family acts as DNA damage-inducing and growth-suppressing genes and plays a tumor suppressor role in targeted therapy (71). GADD45A methylation reduces the inactivation of PI3K-AKT and the radiosensitivity of cervical cancer. MK2206 increased the radiosensitivity of SiHa cells, suggesting that the PI3K-AKT pathway is related to radiotherapy resistance. The overexpression of SKA3 activates the PI3K/AKT signaling pathway, increases the levels of p-AKT, cyclin D1, CDK4, CDK2, p-Rb and E2F1, promotes the proliferation and migration of HeLa cells, and accelerates tumor growth (72). The AKTi (GSK690693) significantly reversed the cell proliferation ability induced by SKA3 in HeLa cells. These findings position SKA3 as both a potential therapeutic target and an independent prognostic biomarker for cervical cancer.

Clinical trials of AKTi in cervical cancer are relatively limited. An investigator-initiated phase II study (NCT01958112) which combining trametinib and GSK2141795 in patients with recurrent cervical cancer enrolled 14 patients (73). The results indicated that 1 patient had unconfirmed PR, 8 patients were SD, 3 patients were PD, and 2 patients were not evaluable. Toxicities were primarily grade 1/2, with 57% of patients experiencing grade 3/4 AEs and 50% experiencing dose reduction. The study was terminated early and the results from these 14 patients didn’t support further development of the combination in cervical cancer.

## 5 Summary

AKT is located at the hub of the PI3K/AKT/mTOR pathway andmediate a variety of biological functions, such as cell proliferation, survival, glucose metabolism, protein synthesis, genome stability, and inhibition of apoptosis. The AKTi-related preclinical and clinical trials showed that it had certain anti-tumor activity in gynecological cancers. Currently, there are many new AKTis under continuous development, and some clinical trials are underway. However, in the process of reviewing these literatures, we found that the efficacy of AKTi still needed to be verified by a large number of animal models and clinical trials, and the research about AEs was relatively limited. The research and development of new AKTis is still worthy of expectation. We listed the following hot topics that should be focused on:

1. AKT protein selectivity. Currently, most AKTis are pan-selective and target all three AKT protein isoforms (AKT1, AKT2, AKT3), which may be a reason for limited clinical efficiency. It is possible to develop inhibitors that target each AKT isoform respectively and explore the anti-tumor activity of each type of AKTi as a monotherapy or in combination with other anti-tumor drugs in the future.
2. Clinical translation of AKTi. Many AKTis are still in preclinical or phase I/II clinical trial. There were no phase III clinical trials for gynecological cancers. The clinical translation of AKTi is expected especially for the patients with PROC.
3. AEs of AKTi. As AKT is involved in multiple biological functions, AKTi may lead to systemic reactions, such as affecting glucose metabolism and uptake, causing liver damage, inflammation, and cancer metastasis. It is expected to improve kinase selectivity, reduce the dosage, and develop clinically effective and safer drugs.
4. Biomarker selection. Screening patients with gynecological cancer who may benefit, identifying relevant biomarkers for detection and tracking, and establishing prognostic models are worth exploring.
